# The Southern Journal of the Medical and Physical Sciences

**Published:** 1853-04

**Authors:** A. M. Hooker, J. F. Johnston

**Affiliations:** [ill]; Indianapolis


[For the Dental Register of the West.]

THE SOUTHERN JOURNAL OF THE MEDICAL AND PHYSICAL SCIENCES.

Mr. Editor:—We have had the privilege of perusing the second number of this Journal, and with such pleasure that we feel constrained to recommend it to all our professional brethren. We hail the appearance of such a periodical as another pleasing evidence that the speciality of Dental Surgery is destined ere long to take its proper position as a legitimate branch of the Healing Art. A journal in which this department is allotted an honorable place by the side of General Medicine, Chemistry and Pharmacy, is indeed a bright omen. Such a periodical, we believe, cannot fail to benefit both the parent science, and its legitimate offspring, dental surgery. Whatever tends to restore to the latter the lost affections of its parent and sister sciences, should be hailed with joy by every member of our profession. On this ground as well as for the ability with which we believe it will be conducted, we hope this periodical will receive the support of all who desire the advance of the interests of dental science. It is an undeniable fact that the importance of a knowledge of medicine to the practitioner of Dental Surgery is too little urged, and that dentistry is practiced too much as a mere mechanical art. This is the grand reason why physicians withhold the hand of fellowship from those who devote themselves to the practice of this speciality. A publication like the present we believe is calculated to remedy this evil, and that too without interfering in the least with periodicals devoted exclusively to dentistry. We believe the time will come when a medical education will be considered indispensable to the successful study and practice of Dental Surgery. If the Southern Journal of Medical and Physical Sciences shall tend to hasten that time, it will be a rich [illegible] to our profession.

A. M. HOOKER.

*[illegible], March 15th,* 1853

---

*Cincinnati, Feb. 19th,* 1853.

Professor John Allen.

*Sir*:—The students of the Ohio College of Dental Surgery, whose names are appended, avail themselves of this opportunity, to acknowledge their obligations to you as their instructor, and to express their warmest thanks for the uniform kindness with which you have fulfilled the more than arduous duties, (at a personal sacrifice,) which, by a combination of circumstances, have devolved upon you.

They are also desirous of manifesting their keen appreciation of the liberality with which you have devoted the result of the effort of years, for their benefit, deeming your patent the "beau ideal" of dental perfection, and they assure you that these kindnesses will not only be gratefully remembered, but serve as a stimulus for the attainment of professional excellence. In conclusion; feeling a deep interest in the prosperity of the institution they trust that no unforeseen circumstances may prevent you from resuming the chair you have so ably filled.

| | |
|---|---|
| J. F. Johnston, | Indianapolis, Indiana. |
| W. N. Eames, | New York City. |
| George Laurie, | Ann Arbor, Michigan. |
| D. W. Rondebush, | Batavia, Ohio. |
| J. M. Lewis, | Marion, Illinois. |
| M. W. S. Kendall, | Cincinnati, Ohio. |
| H. P. Marquam, | Darlington, Indiana. |
| H. A. McDaniel, | Todd Co., Kentucky. |
| J. W. Bradley, | Cincinnati, Ohio. |
| J. Richardson, | " " |
| Joseph Payne, | Gallipolis " |
| H. T. Manlove, | Bambrodque " |
| Calvin King, | Pittsburgh Penn. |
| S. B. Dunlap, | Chillicothe, Ohio. |
| J. E. Jones, | Dayton " |
| James T. Irwin, | Cincinnati " |
| John Lea Milton, | Lexington, Mississippi. |

James Taylor, M. D., D. D. S.

*Editor Dental Register. Dear Sir:*—I forward the enclosed for publication, in conformity with the desire of the Dental Class, who are its signers, with the request that it may apppear in the next number of the "Dental Register."

Very Respectfully, Yours, J. F JOHNSTON, D. D. S.

*Indianapolis, March* 10, 1853.

